# Epigenetic biomarkers and preterm birth

**DOI:** 10.1093/eep/dvaa005

**Published:** 2020-06-14

**Authors:** Bongsoo Park, Rasheda Khanam, Vinesh Vinayachandran, Abdullah H Baqui, Stephanie J London, Shyam Biswal

**Affiliations:** d1 Department of Environmental Health and Engineering, Johns Hopkins Bloomberg School of Public Health, Baltimore, MD 21205, USA; d2 Department of International Health, Johns Hopkins Bloomberg School of Public Health, International Center for Maternal and Newborn Health, Baltimore, MD 21205, USA; d3 School of Medicine, Cardiovascular Research Institute, Case Western Reserve University, Cleveland, OH 44106, USA; d4 Department of Health and Human Services, National Institute of Environmental Health Sciences, National Institutes of Health, Research Triangle Park, NC 27709, USA

**Keywords:** preterm birth (PTB), epigenome, diagnostics, methylation, chromatin

## Abstract

Preterm birth (PTB) is a major public health challenge, and novel, sensitive approaches to predict PTB are still evolving. Epigenomic markers are being explored as biomarkers of PTB because of their molecular stability compared to gene expression. This approach is also relatively new compared to gene-based diagnostics, which relies on mutations or single nucleotide polymorphisms. The fundamental principle of epigenome diagnostics is that epigenetic reprogramming in the target tissue (e.g. placental tissue) might be captured by more accessible surrogate tissue (e.g. blood) using biochemical epigenome assays on circulating DNA that incorporate methylation, histone modifications, nucleosome positioning, and/or chromatin accessibility. Epigenomic-based biomarkers may hold great potential for early identification of the majority of PTBs that are not associated with genetic variants or mutations. In this review, we discuss recent advances made in the development of epigenome assays focusing on its potential exploration for association and prediction of PTB. We also summarize population-level cohort studies conducted in the USA and globally that provide opportunities for genetic and epigenetic marker development for PTB. In addition, we summarize publicly available epigenome resources and published PTB studies. We particularly focus on ongoing genome-wide DNA methylation and epigenome-wide association studies. Finally, we review the limitations of current research, the importance of establishing a comprehensive biobank, and possible directions for future studies in identifying effective epigenome biomarkers to enhance health outcomes for pregnant women at risk of PTB and their infants.

## Introduction

Preterm birth (PTB, birth <37 completed weeks of gestation) is a major global public health problem because of its high incidence rate and associated high morbidity, mortality, and long-term disability [1]. Each year ∼15 million ( 11.1% of all live births) worldwide occur preterm [2]. The incidence ranges from ∼5% in many European countries to 18% in certain South Asian and sub-Saharan African countries [3]. More than 60% of all PTBs occur in South Asia and sub-Saharan Africa [3]. High-income countries are also affected, and the USA is one of the top 10 countries with the largest number of PTBs [3]. In the USA, there are significant and persistent racial, ethnic, and socioeconomic differences in the rates of PTB. In 2018, the rate of PTB in various subpopulations was as follows: African American women (13.6%), American Indian/Alaska Native (11.3%), Hispanic (9.4%), White women (9.0%), and Asian/Pacific Islanders (8.7%) [4]. Preterm infants experience greatly increased mortality. In the USA, 34.3% of all infant deaths were classified as PTB associated, and 95% of PTB-associated deaths occurred in infants born at <32 weeks of gestation and with weight <1500 g [5]. Globally, PTB complications account for ∼1 million deaths or 15.4% of all deaths in <5-year-old children, making them the leading cause of death for children in this age group [6]. PTB is also associated with greater lifetime disability, including reduced cognitive function, and visual and hearing impairment , as well as associated with a greater risk of future health conditions such as neurodevelopmental disorders and cardiovascular diseases [7, 8]. The economic burden and lifetime costs of PTB of a child to the caregiver are substantial [9].

The etiology of PTB is not completely understood because of the complex interplay of genetic, environmental, and host factors. There has been a significant effort to understand the disease mechanisms of PTB with several risk factors identified. Rubens *et al.* [10] has summarized the current state of knowledge and potential etiologic pathways involved in PTB. PTB risk analysis from a Danish family cohort showed more significant maternal genetic effects than paternal effects using historic family medical records [11]. Large cohort genome-wide association studies (GWAS) have described potential causal variants associated with PTB [12]. In addition, environmental exposure (e.g. cigarette smoke, heavy metals) is known risk factors for PTB [12]. Placental dysfunction is another important risk factor for PTB, implicated in major complications during pregnancy such as preeclampsia, fetal growth restriction, and stillbirth [13]. Maternal cardiometabolic disease also contributes to PTB risk. For example, obesity or obstetric factors, such as gestational diabetes and hypertension can increase the risk of PTB [14, 15]. PTB may be associated with enhanced oxidative stress as indicated by increased levels of lipid peroxidation biomarker 8-iso-PGF2α metabolite in urine samples [16]. Malnutrition of pregnant women can also increase the risk for PTB [17]. For example, deficiency of iron, folic acid, or vitamin D during pregnancy may increase the risk for PTB [17]. The alteration of maternal micronutrients (folic acid, vitamin B-12) has been found to increase homocysteine and oxidative stress levels, which may in turn cause epigenetic modifications that contribute to PTB [18].

In high-income countries, substantial reductions in PTB-related mortality have been achieved through improved management and care of women with premature labor and PTB, despite increasing rates of PTB [19]. However, the “Born Too Soon” partnership estimated that 50% of preterm deaths in low- to middle-income countries (LMICs) could be prevented by improving the delivery of feasible interventions, such as the use of antenatal corticosteroids in women with preterm labor [20], antibiotics usage for preterm–premature rupture of membranes [21], management of respiratory distress syndrome, and use of Kangaroo Mother Care [22]. The effectiveness of potential interventions requires early identification of increased risk for PTB. Screening for PTB in resource-limited settings is based on clinical grounds with limited predictive ability and not through ultrasonic or biochemical assessments [13]. Obstetric premature delivery screening approaches include cervical length and fetal fibronectin measurements, though their ability to predict early PTB is limited [23]. Determining PTB-related epigenome markers would help to improve understanding of the underlying molecular mechanisms and could serve as a supplementary diagnostic tool utilizing more accessible surrogate bio-samples.

Table 1 summarizes some recent studies exploring epigenome biomarkers potentially related to PTB, with a focus on investigating blood tissue. We noticed that only DNA methylation has been studied as a primary epigenome biomarker, although other types of epigenome profiles are available. In this review, we will outline recent progress made in the development of epigenome assays and discuss the challenges and opportunities for the potential of epigenomic biomarkers for predicting PTB. The purpose of this review is to describe the currently available technologies and available literature on their application to the study of PTB.

**Table 1:** Selected studies on epigenome biomarkers and PTB

Epigenome	Brief summary	Number of sites	Sample size
DNAm	Fetal leukocyte DNA	29 CpG sites	50 [24]
DNAm	Multi-omics profiles (RNA-seq, WGS, DNA methylation) using maternal blood	72 genes	791 [25]
DNAm	Comparison between cord tissue and blood	10 CpG sites	1019 [26]
DNAm	SLC9B1 methylation, fetal intolerance	4 CpG sites	57 [27]
DNAm	Prenatal smoke exposure, epigenome marker	15 CpG sites	754 [28]
DNAm	Birth weight, epigenome marker	34 CpG sites	711 [29]

## Genetic Susceptibility to PTB

Genetic susceptibility is one of the key factors to determine the risk of PTB. Candidate gene and genome-wide analysis have been widely utilized to understand the role of the genetic background in environmental epidemiology field. For example, candidate genetic markers for PTB or low birth weights, such as ADCY5, CDKAL1, HHEX-IDE, and GCK, have been reported [30]. While several candidate genes have been associated with PTB, lack of replication in different cohorts is a major problem [31]. Efforts to find genetic risk and genetic variants associated with PTB continue, and GWAS) have identified candidate regulatory loci associated with PTB. A recent GWAS revealed additional markers (EBF1, EEFSEC, AGTR2) substantially related to gestational length and as well as PTB [32]. By performing integrated analysis using curated genes and GWAS, Uzun *et al.* [33] reported 15 significant pathways using pathway-based PTB risk analysis (e.g. breast cancer estrogen signaling and oxidoreductase activity). Large cohort samples in GWAS have provided a foundational catalog of genetic variations related to PTB. However, PTB is also highly associated with environmental factors. Incorporating environmental interaction into GWAS can identify novel loci that would be missed in analyses of genetic variants alone [34].

## Environmental Exposures and PTB

Several causes of spontaneous PTB have been identified, including maternal smoking during pregnancy, maternal stress, infectious disease, inflammation, and uterine distension [35, 36]. In addition to smoking, various environmental exposures have been identified as potential risk factors for PTB [37]. Several studies in the USA have identified ambient air pollution, including particulate matter, as a risk factor for PTB [38]. Meteorological conditions have also been implicated [39], raising the possibility that climate change could lead to increased PTB in the future. Martinez-Zamudio and Ha [40] summarized the evidence of increased PTB risk associated with exposure to environmental metals (chromium, arsenic, nickel, and cadmium). For example, arsenic exposure from drinking water can contribute to epigenetic reprograming, resulting in the formation of inflammatory cells and more serious conditions like cancers [41]. Also, arsenic reported to affect birth weight through reduced gestational age and reduced maternal weight gain during pregnancy [42].

Many studies have investigated the mechanisms of one toxicant at a time, though such studies do not accurately represent the complex and real-life exposures to complex mixtures. Animal models serve as an informative approach to potentially identify such complex and interactive molecular mechanisms. Ongoing research by the NIEHS TaRGET II Epigenome Consortium will provide a unique opportunity to examine the effect of various toxicants in the prenatal-exposed mouse model [43]. Maternal exposure can leave epigenetic signatures that share the properties across all tissue type, and this metastable epialleles are another target we can investigate for developing effective epigenome biomarker [44–47].

## Epigenome-Wide Association Studies

DNA methylation has been investigated as a major epigenetic mechanism for PTB; in particular, it has been reported that prenatal level of arsenic leads to changes in DNA methylation in placental tissue [48]. In addition, placental DNA methylation has been associated with cadmium [49]. Recent work from epigenome-wide association studies (EWAS) has shown that many of the prenatal exposures implicated in PTB are also related to changes in DNA methylation in cord blood. This finding has been most consistently demonstrated for maternal smoking in pregnancy, where there is widespread alteration in methylation in newborns [50]. EWAS have also shown reproducible associations between blood DNA methylation in newborns and maternal folate levels [51]. Exposure to traffic-related air pollution [52] and particulate matter [53] during pregnancy has been linked to cord blood methylation. EWAS have found exposure to metals, including lead and mercury, during pregnancy to be related to methylation in cord blood [54, 55]. For example, genome-wide analyses have shown that exposure to arsenic *in utero* has led to DNA methylation changes in multiple loci such as PLA2G2C, loci-SQSTM1, SLC4A4, and IGH [56]. Chromium found in airborne particles [57] or contaminated water causes various epigenetic modifications including DNA methylation [58] and histone modifications [40]. While a number of studies have suggested that the exposure-related methylation differences might mediate the effects of exposures on birth outcomes, the causal epigenomic mechanism remains unclear. For example, it has been shown that maternal smoking [59] complicates the use of mediation analysis to identify causal relationships [60, 61].

## Multi-Omics Resource for PTB Studies

We checked for available multi-omics datasets in Gene Expression Omnibus (GEO; https://www.ncbi.nlm.nih.gov/geo/(1 February 2020, date last accessed)) using the search terms “preterm birth” and “homo sapiens,” and found 53 studies and 3050 datasets (Table 2). For gene expression datasets, Affymetrix or Illumina Bead Array methods have been used generating 1756 transcriptome datasets. In addition, more recently, high-throughput sequencing methods have been employed generating 681 datasets. A total of 2437 transcriptome datasets (mRNA and miRNA) were found to be available for PTB meta-analysis in the NIH GEO repository. For methylation datasets, Illumina 450K arrays have been used in the larger population studies. We found 613 DNA methylation samples from 11 studies. GWAS datasets have been utilized to construct a comprehensive database of single nucleotide polymorphisms (SNPs) related to PTB called dbPTB [62]. Recently, the GWAS Catalog reported 63 associations from 15 studies [63]. There is no available PTB open chromatin analysis (ATAC-seq).

**Table 2:** Available transcriptome and epigenome datasets for PTB studies in GEO datasets

Methods	Descriptions and relevant assays	Number of studies	Number of samples
Gene expression			
Microarray	Affymetrix, Illumina Beadarray	32	1789
mRNA	High-throughput sequencing	9	516
Non-coding RNA			
mi-RNA	High-throughput sequencing or array	9	379
DNA methylation			
Illumina arrays	450K, EPIC arrays	12	692
MeDIP-seq	High-throughput sequencing	1	30
RRBS or WGBS	High-throughput sequencing	1^a^	52
Open chromatin			
ATAC-seq	High-throughput sequencing	None	None

^a^WGBS datasets for prenatal exposure with smoke [64] are available in European Genome-phenome Archive (EGAS00001000455). The resources for WGBS are limited. The above number of the datasets was extracted in February 2020.

We summarized examples of research on genome-wide methylation in newborns to PTB or gestational age (Table 3), and the literature suggests that there are widespread epigenetic effects throughout the genome in PTB. The placenta has been investigated as an associated tissue for PTB, and particular immune cell types (e.g. monocytes and CD4 T cells) have also been studied for potential biomarkers in PTB diagnostics. Although the genetic variation and epigenome changes are not completely understood at the mechanistic level, and further investigations are needed to provide effective treatment and health management.

**Table 3:** Examples of DNA methylation studies using multiple tissue types

Tissue type	Sample size	Brief study description
Placenta	480	PTB, birth weight and gestational age [65]
Placenta	206	Maternal tobacco smoke and gestational age [66]
Placenta	158	Locus-specific, MTR gene [67]
Placenta, cord blood	83	Preeclampsia, circadian clock [68]
Cord blood	1753	Gestational age [69]
Cord blood	397	PTB, birth weight [70]
Cord blood	181	PTB and infection [71]
Cord blood	141	PTB and gestational age [72]
Cord blood	83	Very PTB [73]
Cord blood	73	PTB and infection [71]
Cord blood	50	PTB, African American [74]
Cord blood	50	PTB, gestational age [24]
Amnion	121	PTB and labor [75]
Blood spots at birth	24	PTB, effect of gestational age and long-term legacy [76]
Maternal leukocytes	24	PTB [77]
Blood	144	PTB, twin study [78]
Maternal, cord blood	300	PTB, African American [79]
Buccal cell DNA	536	Neurobehavioral variation, preterm infants (29 CpG) [80]

PTB, preterm birth.

## Epigenetic Reprogramming as a Major Diagnostic Source for PTB

The epigenome encompasses DNA methylation, histone modifications, high-level chromatin structure, and chromatin accessibility [81]. The most well characterized and highly studied epigenome is DNA methylation at cytosine residues [81]. DNA methylation has useful features for biomarker development. DNA methylation is covalent methylation that is biochemically stable. The most commonly assayed epigenetic marker in current epidemiological research at the population level is DNA methylation [82]. DNA methylation can be captured by a relatively low cost of Illumina DNA methylation arrays (e.g. 450K and EPIC) and sequencing-based methods (e.g. whole-genome bisulfite sequencing).

Histone markers, the post-translational modifications are the second epigenome marks on the N-terminal-exposed tails of histones [83]. Histones (H2A, H2B, H3, and H4) are highly conserved and positively charged proteins that are packaged with the DNA to form nucleosomes [84]. A total of 147 bp of DNA wraps around two copies of each histone molecule [85]. Packaging of the DNA not only serves to make the genome more compact but also to regulate the accessibility of *cis*- and *trans*-regulators for the DNA-templated processes such as replication, transcription, recombination, and repair. Covalent modifications in the DNA, N-terminal tails of histones, and to some extent, heritable non-coding RNA, are termed as epigenetic modifications [81, 83]. Most of these covalent modifications change the nucleosome composition, which in turn alters gene expression without any change in the underlying DNA.

The chromatin immunoprecipitation with parallel DNA sequencing (ChIP-seq) is used to profile histone modifications and transcription factor-binding sites. It requires a large amount of input material and is therefore difficult to deploy in large cohort studies. In addition, there are substantial sample-to-sample variations in both transcription factor-binding sites [86] and histone modifications [87]. Due to this reason, there are very few examples of large-scale population-level studies using ChIP-seq. The recent advancement in molecular assay enables low input ChIP-seq to be used for particular biological assays involving a small number of cells [88]. The insulator protein CTCF is cell-type-specific; therefore, it has been used for cancer cell profiling. Interestingly, the plasticity in CTCF-binding occupancy is also highly correlated with changes in DNA methylation [89].

It is challenging to use the classical assay for chromatin accessibility, such as DNase hypersensitivity. This issue has been largely overcome due to the recently developed ATAC-seq assay for chromatin accessibility, which has low input requirements and an easy-to-use protocol [90]. Since its introduction, this assay has been used in several population-level studies [91]. Finally, the study of the 3D structure of cells and chromatin has gained increased attention in the past years. The 4D Nucleome Project [92] is dedicated to exploring the structure of cells and chromatin. High-level chromatin structure may be a source to elucidate the most important epigenetic markers once the technology is matured for commercialization of a more cost-effective method for large-scale population-level studies. This emerging molecular technology is in the early stage, and the analytical method is being developed.

## Applying Epigenome Biomarkers to Identify Prenatal Complications

DNA methylation can occur in diverse regulatory regions: promoters, enhancers, and insulators. A recent study has shown the significance of DNA methylation in regulating the transposable elements, including LTRs, LINEs, SINEs in response to environmental exposure [93]. In general, gene expression is negatively correlated with hypermethylation in the promoter. DNA methylation in CTCF-binding sites (four CpG sites in the motif) can disturb regulation of surrounding genes, causing epigenetic transcription reprograming. As noted above, DNA methylation at CpG sites is a stable and sensitive biomarker for early detection of the impact of some environmental toxins. As the human embryo and fetus are susceptible to toxins in the pregnant mother’s environment [94], epigenome markers can be investigated to understand diseases induced by environmental exposures (phenotype–epigenome association).

Understanding the molecular mechanisms, including epigenetic reprograming, is a critical step to gain insight into complex prenatal disease etiology. Toxins, such as arsenic, bisphenol A, lead, tetrachlorodibenzodioxin, diethylhexyl phthalate, and particulate matter (particles <2.5 nm), are being investigated in a consortium effort by the Toxicant Exposures and Responses by Genomic and Epigenomic Regulators of Transcription (TaRGET) program funded by NIEHS [43]. In efforts to identify reliable epigenome markers for PTB, the consortium and other researchers have been particularly investigating blood-driven biomarkers. For example, researchers have used neonatal blood to successfully identify CpG sites related to PTB [76, 95]. Interestingly, many of these CpG sites are located in genes related to the Notch signaling pathway. The TaRGET program supports research on multiple toxins and associated disease outcomes by applying the state-of-the-art multi-omics technology such as Whole-genome Bisulfite Sequencing (WGBS), ATAC-seq, ChIP-seq, and RNA-sequencing.

## Potential Advantages of WGBS Over Microarray-Based Methods

Environmental scientists and epidemiologists have often utilized microarray-based DNA methylation assays (e.g. HumenMethylation27, HumenMethylation450, and Illumina EPIC array) due to relatively lower cost compared with whole-genome sequencing methods. While WGBS is a standard method for checking global methylation patterns in the genome, it remains expensive, and thus, it is not practical to use WGBS for population-level studies. In addition, because of the high cost, it is tempting to use low coverage in larger population studies creating possibilities for false-positive results given the paucity of studies with sequencing data for replication. In contrast, the Illumina EPIC array is affordable, and as a consequence, it is widely used in population-level studies enabling meta-analysis and replication.

WGBS can provide much higher resolution compared to the EPIC array, providing ∼28M CpG sites in the human genome. WGBS can detect methylation levels at every single CpG site, which is important to the field of environmental epigenetics because methylation differences associated with environmental exposures are not uniformly distributed across the genome [96]. While this reflects the need for larger sample sizes and better assessment of exposures to reduce measurement error for exposures that are less prevalent or have smaller effects, it is possible that these exposures impact CpGs not covered by existing platforms. WGBS is also an unbiased assay compared with reduced representation bisulfite sequencing (RRBS) [97] or eRRBS [98], which are C/G restriction enzyme-based assays. Restriction enzyme digestion can create biased sequencing fragments, which can produce difficulties to examine reproducibility and to detect consistently differential methylation regions. Furthermore, WGBS provides more information regarding regulatory regions (Table 3).

Nonetheless, WGBS does have some limitations. For example, WGBS alone cannot distinguish between methylation (5 mC) and hydroxymethylation (5 hmC) sites. But the primary limitation of WGBS is the greater difficulty of analysis of sequencing data compared to that of microarray-based technologies.

As sequencing technologies continue to advance, sequencing costs are expected to decline, and expertise in analyzing sequencing will continue to increase. Using the recently released S4 flow cell of Illumina’s NovaSeq 6000, it is possible to process more than 30 WGBS samples in one lane, generating considerable savings. Environmental epigenetic research aims to sequence 30× coverage for WGBS. However, it is suggested to be a cost-effective approach to sequence 5–15× average coverage using more biological replicates (increasing number of samples) [99]. Portable, lower-cost sequencing devices such as Oxford Nanopore GridION could be effective for future diagnostics [100]. However, the accuracy level of these portable devices is poor compared to the Illumina high-throughput sequencing machines. We nonetheless anticipate that the accuracy of portable devices will improve in the future. A portable or small-size sequencer combined with effective biomarkers could be useful for quick diagnostics.

## Chromatin Accessibility and Population-Level ATAC-seq

To perform comprehensive diagnostics using epigenome markers, it is also possible to examine open chromatin signatures (or accessibility) using blood samples. ATAC-seq is widely used for determining open chromatin regions in the genome, incorporating hyperactive Tn5 transposase into accessible regions of chromatin [101]. The open chromatin regions are important candidates as transcription-binding sites (e.g. sequence-specific or general transcription factors) and potential regulatory regions. ATAC-seq is specifically designed to effectively sequence samples consisting of a relatively small number of cells; for instance, blood ATAC-seq requires only 5000 cells [102]. Thus, it has become a popular tool in epigenomics research and can be used in population-level epigenome studies [103]. Omni-ATAC is an improved ATAC-seq protocol that allows chromatin accessibility profiling [104]. This advanced protocol significantly enhances signal-to-background ratios; therefore, the information contained under the peaks (bioinformatically predicted open chromatin regions) is higher. Moreover, this protocol can be applied to archival frozen blood and tissue samples, making it especially suitable for epigenetic epidemiological studies.

TaRGET consortium has developed a robust quality control matrix for high-quality ATAC-seq assays, and open-source scripts are available to the scientific community (http://www.targetepigenomics.org/). In particular, the bioinformatics group has been currently generating visualizations of sequenced ATAC-seq reads in a genome browser for validation and analysis of the binding of reads at different functional regions, including enhancers, transcription starts sites, gene body, and transcription end sites of genes (active or inactive), or at intergenic regions. The profiles of open chromatin could be classified using diverse genomic features as described in Table 4. In epigenetic diagnostics, it would be important to keep recordings, software version, and the reference datasets used for accurate validation.

**Table 4:** Summary of reference features widely used in epigenome analysis

Genomic features	Descriptions and relevant assays
Transcription start sites	Bivalent/poised TSS, flanking TSS, CAGE analysis [105]
Promoter	1 kb from TSS, enriched marks: H3K4me1, H3K4me3
Enhancer	H3K4me1, H3K27ac, p300 ChIP-seq
Insulator	CTCF-binding sites, Hi-C, Hi-ChIP
CpG island, sites	28 million CpG sites (human), 21 million CpG sites (mouse)
CpG shore	Surrounding area (2 kb) from CpG islands
Exon	mRNA, lncRNA, GENCODE prediction, RNA-seq
Intron	Transcript, alternative splicing, GENCODE prediction, RNA-seq
Repeated genome	Transposable elements: LTRs, LINEs, SINEs
Repressed marks	PolyComb, heterochromatin, H3K27me3 ChIP-seq
Activated genes	Transcriptome, PolII, H3K36me3 ChIP-seq
TFBS	CTCF-binding sites, sequence-specific factor bindings (CG rich)
MicroRNA	Non-coding RNA, NIH epigenome roadmap [106]

## Cell-free DNA and Nucleosome Signatures

Histones (H2A, H2B, H3, and H4) proteins are packaged with DNA to form nucleosomes [84], positioned in the specific distance from each other. Nucleosome positioning indicates that nucleosomes are located in the genomic DNA sequence, and it varies between cell types and disease status. Recently, cell-free DNA (cfDNA), which is typically a short fragment of 200 bp, [107], has been isolated from circulating blood plasma and utilized for detecting nucleosome signatures of cancer cells [108]. The size distribution of cfDNA is 147 bp (nucleosome size), and sometimes, it includes the size of a linker histone (167 bp) [109, 110].

Interestingly, cell-free fetal DNA has been detected in the plasma of a pregnant woman [110]. Dugoff *et al.* [111] assessed the connection between the first and second trimesters of cfDNA and confirmed that enhanced concentrations of fetal fractions during gestation (14.1–20.0 weeks) were substantially associated with enhanced incidence of PTB. van Boeckel *et al*. [112] investigated cell-free fetal DNA and spontaneous PTBs, and they found potentially pro-inflammatory properties of cell-free fetal DNA. Snyder *et al*. [108] hypothesized that cfDNA is the detritus of cell death, and the boundaries of cfDNA could reflect patients’ nucleosome signatures in target tissues, making cfDNA as an important epigenetic surrogate material. Nucleosomes are favorably positioned near various regulatory regions such as transcription start sites [113] and exon–intron boundaries [114, 115]. By creating nucleosome maps for healthy and diseased target and surrogate cells in experimental studies, potential disease risks can be categorized.

## The Challenges of Cell Heterogeneity for Developing Epigenomic Biomarkers

Like most tissues, blood is a heterogeneous mixture of different cell types, and human blood cells are constantly being generated in the bone marrow [116–118]. Hematopoietic stem cells are differentiated and divided into common myeloid progenitors and common lymphoid progenitors [116]. Later, common myeloid progenitors differentiate into megakaryocytes, erythrocytes, mast cells, and myeloblasts [116]. Due to the complex mixture of the resulting blood tissues, researchers sort the cells into the specific immune cell (e.g. monocyte) and examine the cell-type-specific DNA methylation profiles. Therefore, it is necessary to create an integrative deconvolution epigenome analysis pipeline. Most studies of epigenetic changes and PTB to date (Table 3) have used whole blood, and thus statistical methods were used to adjust for cell composition, but to date, reference databases are small, and methods are evolving. In addition, some of these studies were done prior to the availability of newborn reference panels. In addition to the usual issues of estimating cell-type composition, studies of gestational age in relation to newborn blood DNA are complicated by the presence of nucleated red cells that decrease with longer gestation. Reference-free methods do not require prior cell-type-specific knowledge [119, 120] but run the risk of removing some of the true variations under study. An intermediate approach is a mixed model such as a semi reference-free method [121]. In contrast, reference-based methods require a database of cell-type-specific epigenome profiles [122], and these are limited for newborns. Larger reference panels would advance the field.

## Emerging Single-Cell Technologies and Computational Methods

Researchers have developed various single-cell-based epigenome assays including single-cell methylation [123], single-cell ATAC-seq [124], and single-cell RNA-seq [125]. These assays effectively address the cell heterogeneity issue, providing comprehensive epigenome or transcriptome profiles. Currently, these cutting-edge technologies have not been sufficiently developed for diagnostics usage, and these are not cost-effective, but in the future, due to advancement in this field, these technologies could be applied to many clinical and biomedical applications. The single-cell level epigenome is still under development, and it is not practical to apply it to the population-based epigenome biomarker development. In fact, effective biomarkers may not be the actual epigenetic mediators or modifiers involved in the molecular mechanisms of the disease. To find reliable epigenome biomarkers, we need large cohort studies to generate robust epigenetic profiles. Once such a large cohort omics dataset is produced, effective computational methods, and models could be derived. Based on the information on the genetic variation, SNP, and family history using electronic medical record, we can incorporate the additional epigenome profiles such as DNA methylation, histone modification, chromatin accessibility, and cell-free nucleosome profiles. Combining large datasets requires computationally intensive analysis pipeline and sophisticated analytic methods. Emerging machine learning tools and artificial intelligence can facilitate the robustness of the prediction power for PTB.

## Biobank for Exploring Epigenomic Marks of PTB

Around the world, there have been several cohorts on pregnancy and childhood outcomes. The global Pregnancy and Childhood Epigenetics (PACE) Consortium, which connects over 39 studies in different countries [126], has examined associations between DNA methylation, primarily using the Illumina 450K array, and various prenatal exposures including smoking, air pollution, maternal body mass index, and alcohol intake [50, 52, 53, 127] and health outcomes including birth weight, maternal hypertension, and childhood asthma [128–131]. Combining data from many studies using meta-analysis has the advantage of generating large sample sizes and also provides built-in replication, reducing false-positive findings. PACE publications to date have used DNA methylation data primarily from newborns and children. Some PACE studies have also DNA methylation data from pregnant women, but no PACE meta-analyses have yet focused on these data. PACE cohorts are predominantly based in high-income countries. Although the burden of PTB is much higher in LMIC, there is hardly any epigenetic research to identify risks and markers of PTB from these countries. Efforts to pursue research in LMIC have been hindered due to a lack of functional biobanks in those settings. Recently, a number of biorepositories in pregnancy cohorts have been established in LMICs which will facilitate discovery of epigenetic markers of PTB [132, 133]. These biobanks can create new opportunities to conduct research to identify markers of PTB for LMICs.

## Conclusions

The development of effective biomarkers that can predict increased risk of delivering PTB during pregnancy is essential. Genetic variation, while useful, will not be sufficient to explain the risks of PTB due to environmental factors. Epigenome signatures are dynamically changed as a response to environmental exposure. Thus, in addition to understanding the genetic variations, we need to develop epigenome biomarkers that can cover genome-wide epigenome signatures such as DNA methylation and chromatin accessibility. Multi-omics approaches to PTB will also be useful as the availability of multi-dimensional datasets increases. These datasets include transcriptome, epigenome, proteome, and metabolomics. In addition to multi-omics datasets, we need to continue developing the standardized and effective way to measure maternal stress, socioeconomic status, ethnic background that contribute the increase risk of PTB. In fact, the mechanisms of PTB are complex and poorly understood. Developing highly predictive biomarkers will enable public health interventions and also increase understanding of the underlying mechanisms. For developing such biomarkers, a large number of samples from prospective cohorts will be needed. Identification of omics markers in target tissue such as placental samples may be easier but not sufficient. It will be important to identify pregnant women at risk of PTB early in pregnancy so that they can be preferentially managed. Therefore, any biomarkers identified in placenta will have to be identified on surrogate tissue samples e.g. blood or even in samples that can be collected non-invasively (e.g. urine, saliva). PTB remains a challenge in the USA and globally as it contributes to health disparities. We encourage the translational bioinformatics and genomics community to contribute to deciphering the complex signatures of epigenome biomarkers and understanding their role in PTB.

## Funding

S.B. is partly supported by NIEHS (U01ES026721). R.K. and A.H.B. are part of Bangladesh AMANHI study funded by the Bill and Melinda Gates Foundation and coordinated by the World Health Organization. R.K. and A.H.B. are also part of Multi-omics for Mothers and Infants (MOMI) consortium funded by the Bill and Melinda Gates Foundation. S.J.L. is supported by the Intramural Research Program of the NIH, National Institute of Environmental Health Sciences (NIH ZO1 ES49019).

*Conflict of interest statement.* None declared.
